# New Stimulation Device to Drive Multiple Transverse Intrafascicular Electrodes and Achieve Highly Selective and Rich Neural Responses

**DOI:** 10.3390/s21217219

**Published:** 2021-10-29

**Authors:** Thomas Guiho, Victor Manuel López-Álvarez, Paul Čvančara, Arthur Hiairrassary, David Andreu, Thomas Stieglitz, Xavier Navarro, David Guiraud

**Affiliations:** 1 Camin Team, National Institute for Research in Computer Science and Automation (Inria), 34095 Montpellier, France; arthur.hiairrassary@neurinnov.com (A.H.); david.andreu@neurinnov.com (D.A.); david.guiraud@inria.fr (D.G.); 2 Camin Team, Laboratoire d’Informatique, de Robotique et de Microélectronique de Montpellier, Université de Montpellier, 34095 Montpellier, France; 3 Department of Cell Biology, Physiology and Immunology and Institute of Neurosciences, Universitat Autonoma de Barcelona (UAB), 08193 Bellaterra, Spain; vmlopal@gmail.com (V.M.L.-Á.); xavier.navarro@uab.cat (X.N.); 4 Centro de Investigacion Biomedica en Red en Enfermedades Neurodegenerativas (CIBERNED), 08193 Bellaterra, Spain; 5 Laboratory for Biomedical Microtechnology, Department of Microsystems Engineering—IMTEK, University of Freiburg, 79110 Freiburg, Germany; paul.cvancara@imtek.uni-freiburg.de (P.Č.); Thomas.Stieglitz@imtek.uni-freiburg.de (T.S.); 6 Neurinnov, 34095 Montpellier, France

**Keywords:** stimulation selectivity, stimulation device, intrafascicular electrode, peripheral nerve, neuroprosthesis

## Abstract

Peripheral Nerve Stimulation (PNS) is a promising approach in functional restoration following neural impairments. Although it proves to be advantageous in the number of implantation sites provided compared with intramuscular or epimysial stimulation and the fact that it does not require daily placement, as is the case with surface electrodes, the further advancement of PNS paradigms is hampered by the limitation of spatial selectivity due to the current spread and variations of nerve physiology. New electrode designs such as the Transverse Intrafascicular Multichannel Electrode (TIME) were proposed to resolve this issue, but their use was limited by a lack of innovative multichannel stimulation devices. In this study, we introduce a new portable multichannel stimulator—called STIMEP—and implement different stimulation protocols in rats to test its versatility and unveil the potential of its combined use with TIME electrodes in rehabilitation protocols. We developed and tested various stimulation paradigms in a single fascicle and thereafter implanted two TIMEs. We also tested its stimulation using two different waveforms. The results highlighted the versatility of this new stimulation device and advocated for the parameterizing of a hyperpolarizing phase before depolarization as well as the use of small pulse widths when stimulating with multiple electrodes.

## 1. Introduction

Neuroprostheses are active medical devices aiming at restoring impaired physiological functions via the injection of electrical charges close to excitable cells, especially neurons. Among the disorders that can benefit from these technologies is Spinal Cord Injury (SCI) [1]. Characterized by a breakdown in supraspinal communication, SCI invariably results in alterations of the sublesional functions and leads to visceral (sexual, urinary etc.), sensory (anesthesia, pain) and motor (paraplegia, tetraplegia) deficits that have a dramatic impact on a patient′s quality of life [2,3].

The preservation of excitatory properties of the sublesional tissue after SCI assists in configuring approaches to interface with the preserved cells so as to activate them vis electrical stimulation and to promote functional rehabilitation. Electrical stimulation thus implies a broad spectrum of clinical applications for functional restoration ranging from breathing facilitation by phrenic nerve stimulation [4] to bladder [5,6], bowel [7,8], sexual and motor [9,10,11] functions. Concerning the latter, several modalities of stimulation are currently investigated according to electrodes positioning—and design—distinguishing the spinal, intramuscular, epimysial, surface and Peripheral Nervous System (PNS) approaches. PNS is of particular interest as it overcomes most muscle stimulation constraints, i.e., the activation of many muscles from a single electrode (limiting surgical complexity and technical failures) and finer stimulation settings (reducing power consumption and opening new possibilities for hardly accessible muscles). Indeed, many muscles are controlled by the same nerve, and the application of targeted stimulations should, in theory, enable the selective activation of several muscles, since the nerve fibers controlling a specific muscle are grouped in the same fascicle [12,13].

Concerning afferent pathway stimulation, sensory feedback restoration is a potential application for such a technology as it requires selective stimulations to evoke specific sensations. Multicontact cuffs [14,15] and multicontact intrafascicular electrodes [16] already successfully elicited a large range of phantom hand sensations, providing richer and bidirectional hand prosthesis interfaces. However, despite these advantages, the potentially limiting factors of the electrical stimulation in comparison with physiological activation still need to be considered before PNS stimulation becomes a standard procedure [17].

Electrode design is of high relevance in PNS efficiency since it influences the paths of current dissipation, and the excitation threshold and spatial selectivity vary with the distance of the fibers to the stimulation sites. Nerve fibers that are far from the electrode are only slightly affected by stimulation while the nearest fibers are preferentially depolarized. Due to the peripheral nerve structure, two stimulation modalities were considered—i.e., stimulation using electrodes with contacts in close connection with the nerve surface [18] or via electrodes directly inserted through the nerve [19]. The first mentioned class belongs to the “cuff electrodes” family or to cuff electrode-related technologies (e.g., the nerve flattening FINE [20]) and preferentially activates fibers at the periphery while the second generation of the aforementioned electrodes belongs to the family of “intrafascicular electrodes” and targets the fibers within the nerve. The latter are either inserted longitudinally (Longitudinal IntraFascicular Electrode or LIFE [21]) or transversally (Transverse Intrafascicular Multichannel Electrode or TIME [22,23,24]) to specifically target fascicles in superficial and deeper nerve areas.

Another limitation in PNS spread is linked to disparities in the activation thresholds of the different nerve fiber populations. Depending on their diameter and their conductive properties (presence/absence of myelin sheath), nerve fibers become more or less sensitive to stimulation. Thus, the large diameter-myelinated fibers are preferably activated at low currents. In order to manage this phenomenon, numerous simulation studies investigated the impact of stimulation parameters—i.e., the pulse width (PW), intensity (I), frequency (f)—on the relative recruitment of fiber populations using computational models [25,26,27,28,29]. Alongside these simulations, experimental techniques for obtaining a normal recruitment order have been described in the literature [30]. Among them, an anodal block is an extensively studied procedure that relies on the hyperpolarization of nerve fibers near an anode to selectively block evoked-action potentials from larger fibers [31]. In the same way, subthreshold depolarizing prepulses that are close to the excitation threshold exploit the accommodation of the fiber membrane and the inactivation of voltage-dependent sodium channels to influence the recruitment order [32]. The C-fibers’ selective activation was also investigated using slowly rising ramp pulses [33] while the high frequency block method uses rectangular or sinusoidal pulses of frequency 3–5 kHz to block activation of the nerve by preserving it in a refractory period [34].

According to these guidelines, the optimization of PNS strategies requires the refining of stimulation parameters and the delivery of complex waveforms through multiple contacts dispatched among several multichannel electrodes. This implies a complex waveform design on the synchronous output and an increasing number of wires between the stimulus generator and the electrode contacts (poles). A programmable multidimensional stimulus waveform provides the opportunity to conduct research on artificial-to-natural interfaces in order to achieve an efficient and minimally aggressive activation [35,36,37,38]. In contrast, the vast majority of the solutions that have been tested on humans have been based on centralized implants through which the wires output to monopolar or bipolar electrodes [39,40,41]. Two exceptions can be noted: BION technology, where bipolar stimulation is provided by injectable autonomous units [42], and the LARSI project, which is aimed at multipolar stimulation [43] localized to the sacral roots.

In this study, we present a new portable multichannel stimulation device—called STIMEP—designed to automatically deliver sequences of complex stimulation patterns for up to four electrodes simultaneously. We tested this device in vivo by stimulating rats’ sciatic nerve with TIME electrodes along three modalities. Firstly, we finely tuned the stimulation parameters—intensity and pulse width—and investigated their impact on nerve fibers within a single fascicle; secondly, we implemented simultaneous stimulations of several TIMEs for the same nerve and studied spatial selectivity; and lastly, we tested two different stimulation patterns. We used these protocols to ensure stimulator performances and safety but also to gain further insights regarding the potential of TIME electrodes for optimized PNS strategies.

## 2. Materials and Methods

### 2.1. General Description of the Stimulation Device—STIMEP Platform

STIMEP is a wearable neural stimulator designed and developed by the CAMIN team (former DEMAR team, INRIA/University of Montpellier) in association with the Axonic company (Sophia Antipolis, Vallauris, France). The hardware and embedded software architectures [44] were developed in compliance with European Union directives (90/385, 93/342 directives and EN 62304 before the application of the new EU regulation MDR2017/745) for active medical devices so as to permit its use for human clinical trials. STIMEP is piloted by means of the SYNERGY neurostimulation software (Camin team, INRIA, Montpellier, France).

**Figure 1:**
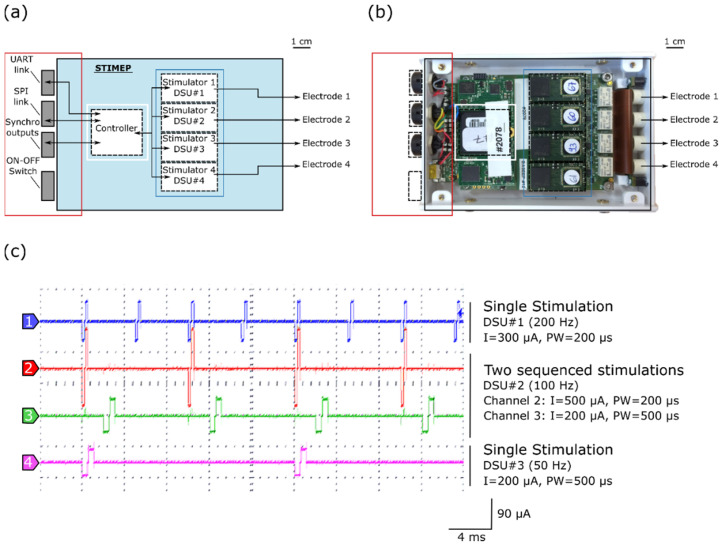
STIMEP architecture and bench testing. (**a**) Diagram of STIMEP hardware and (**b**) picture of the corresponding device at the same scale. (**c**) Coordinated DSU stimulations with different frequencies, intensities and pulse widths (oscilloscope screenshot).

The STIMEP platform allows for the control of 64 contacts (56 active sites and 8 ground sites) of implanted electrodes, divided across 4 insulated ports of 14 capacitive coupled active outputs and 2 direct-coupling references. The stimulation device consists of a controller (SOC device) that drives and coordinates the 4 distributed stimulation units (DSU) able to drive up to 4 TIME devices simultaneously and independently (Table 1, Figure 1a,b). The controller embeds and executes a set of functions [45], for instance, those dedicated to “Contact Check” (qualitative impedance measurement), “Threshold Determination” (increasing stimulation to determine the sensation threshold), “Therapy” (for protocol-dependent multisite stimulation), etc., and controls the set of DSU depending on the given function. Each DSU is composed of an analog ASIC stimulation front-end (CAFE24 chip—designed by the company MXM under CAMIN licensing [WO2006/027473]) and a digital architecture based on FPGA that allows for communication functions, the sequencing of stimulations, the control of stimulation profiles and safety monitoring by means of reference models (ensuring contact and nerve integrity). The SYNERGY software was developed to configure and pilot the STIMEP controller, thus leading to the generation of complex stimulation patterns by adjusting both the stimulation parameters and waveforms on many independent channels (depending on the executed function).

Each DSU embeds a scheduler, thereby allowing for sequencing stimulations, in accordance with a given frequency (Figure 1c). The remote configuration and control of the (set of) DSU is ensured by the controller, depending on the executed function, for coordinating and modulating stimulations. The controller also generates synchronization signals to trigger the recording devices.

### 2.2. In Vivo Validation of the Stimulation Device and Selectivity Study

#### 2.2.1. Experimental Set-Up

Surgical procedures were performed in 2 female Sprague-Dawley rats (280–300 g) under pentobarbital anesthesia (40 mg/kg intraperitoneal i.p.) and in accordance with protocols approved by the Ethical Committee of the Universitat Autonoma de Barcelona. The right sciatic nerve was exposed at mid-thigh and gently dissected from the surrounding tissue between the sciatic notch and the knee. The TIMEs were implanted with the aid of a dissecting microscope to ensure the correct positioning of the device. The needle attached to the central axis of the TIME device was transversally inserted into the sciatic nerve proximal to its trifurcation at the knee before transferring the stimulation sites into the nerve. Since interfascicular selectivity—i.e., the selective activation of fibers laying in separate nerve fascicles—is relatively easy to assess by positioning different contact sites in the selected fascicles [22], we mainly focused our analysis on intrafascicular selectivity. As the TIMEs that were used were originally designed for human trials, only two contacts were positioned within the rat sciatic nerve concurrently (i.e., one contact in the left side and one in the right side).

The required stimulation was provided by a STIMEP stimulator and configured using SYNERGY software. Different stimulation paradigms were tested while the electromyographic signals were acquired. Three muscles that were innervated by the sciatic nerve were monitored throughout the procedure. The compound muscle action potentials (CMAPs) from the Plantar Interossei (PL), Gastrocnemius (GM) and Tibialis anterior (TA) muscles were recorded using pairs of EMG needle electrodes that were directly inserted in each muscle.

#### 2.2.2. TIME Implants

The TIME implants were developed and designed by the Laboratory for Biomedical Microtechnology (Department of Microsystems Engineering—IMTEK, University of Freiburg, Freiburg im Breisgau, Germany) [23]. The implants consist of an L-shaped thin-film part that is connected, via the microflex interconnection technique (MFI) [46], to a screen-printed ceramic. Opposite to the MFI, a custom made 10 cm long cable was soldered. The cable consists of 16 helically wound MP35N wires which are insulated by polyesterimide (PEI). The wires are protected by a medical grade silicone rubber tubing (Helix Medical Europe GmbH, NuSIL MED-4750, Kaiserslautern, Germany), which is also contains silicone rubber. The cable terminates in a 16 channel nano neuroconnector by Omnetics (NCP-16-DD, Omnetics Connector Corporation, Minneapolis, MN, USA) for the electrical connection with the stimulation device. The exposed parts that will be implanted are additionally covered with medical grade silicone rubber (MED 1000, NuSIL Technology LLC, Carpintaria, CA, USA).

The thin-film element uses polyimide as the substrate and insulation material. It incorporated 16 active sites, including 14 independent stimulation sites of 80 µm in diameter and 2 ground sites (GND) of 109 hexagonal arranged contacts (80 µm in diameter; total area of about 0.55 mm^2^) which are connected amongst themselves under the polyimide. The polyimide is fabricated through standard lithographic processes in a class 5 cleanroom. Platinum is used as the conducting material for the tracks. To ensure good adhesion to the polyimide, silicon carbide is used as an adhesion promoter. In order to increase the charge injection, a highly porous sputtered iridium oxide film is used on the active sites. The openings are obtained via reactive ion etching.

**Figure 2:**
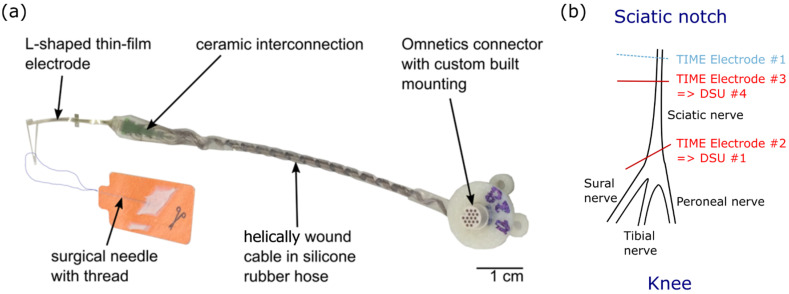
Transverse Intrafascicular Multichannel Electrode (TIME) and implantation procedure. (**a**) Photograph of the TIME-4H implant. Each TIME consists of 14 independent stimulation contacts and 2 ground sites. (**b**) Implantation of TIME devices into the sciatic nerve proximal to its trifurcation at the knee especially for protocol 2.

Its design (version TIME-4H) is symmetric, so that after the peeling of the thin-film electrode from a silicon wafer (used for fabrication), it can be folded in the assembly procedure from a U shape to an L shape (similar to previous publications for the TIME-3H [47]). This allows for the integration of a surgical needle with a suture sling (Prolene, EH7900G, Johnson & Johnson Medical Co., New Brunswick, NJ, USA), which facilitates the transverse implantation through the targeted nerve. Once folded, the contacts are distributed across two compartments positioned on each side of the electrode (7 stimulation sites and 1 ground). For the purpose of implantation, the stimulation sites—separated by a pitch of 0.75 mm—are inserted transversally within the nerve while the grounds are disposed distally from the implantation site in parallel—but still in close contact—to the nerve surface. The pitch of the left and right active site is displaced, relative to one another, to half the pitch (0.375 mm) to facilitate multi-fascicles stimulation (Figure 2a).

#### 2.2.3. Stimulation Paradigms

Prior to the animal experiments, we designed three original protocols to ensure both STIMEP compliance with prerequisite technical and safety specifications and to assess advanced TIME-dedicated PNS paradigms. First, we performed finely tuned current intensity scanning with different pulse widths using contacts in a single fascicle. We automatically generated the strength duration curve and used these data to identify the most energy efficient configurations around the chronaxie. In the second step, we implanted two electrodes within the same sciatic nerve along different axis and ensured the STIMEP ability to drive several DSUs simultaneously. We further capitalized on this opportunity to explore the potential of a multi-TIME device approach and aimed to improve spatial selectivity by targeting different fascicles and/or portions of the nerve. Finally, we tested two different stimulation patterns by reversing the polarity of the original symmetric biphasic pulse (bipolar configurations—one of the two references applied in association with an active contact to enable current flow)—i.e., the original stimulation with a depolarizing pulse, followed by an interpulse of 100 µs [48] and a repolarizing phase, while the second pattern was constituted by a hyperpolarizing pulse followed by an interpulse (100 µs) and a depolarizing phase.

- Protocol 1: Scanning with intensity for different pulse widths using a single TIME implant

**Figure 3:**
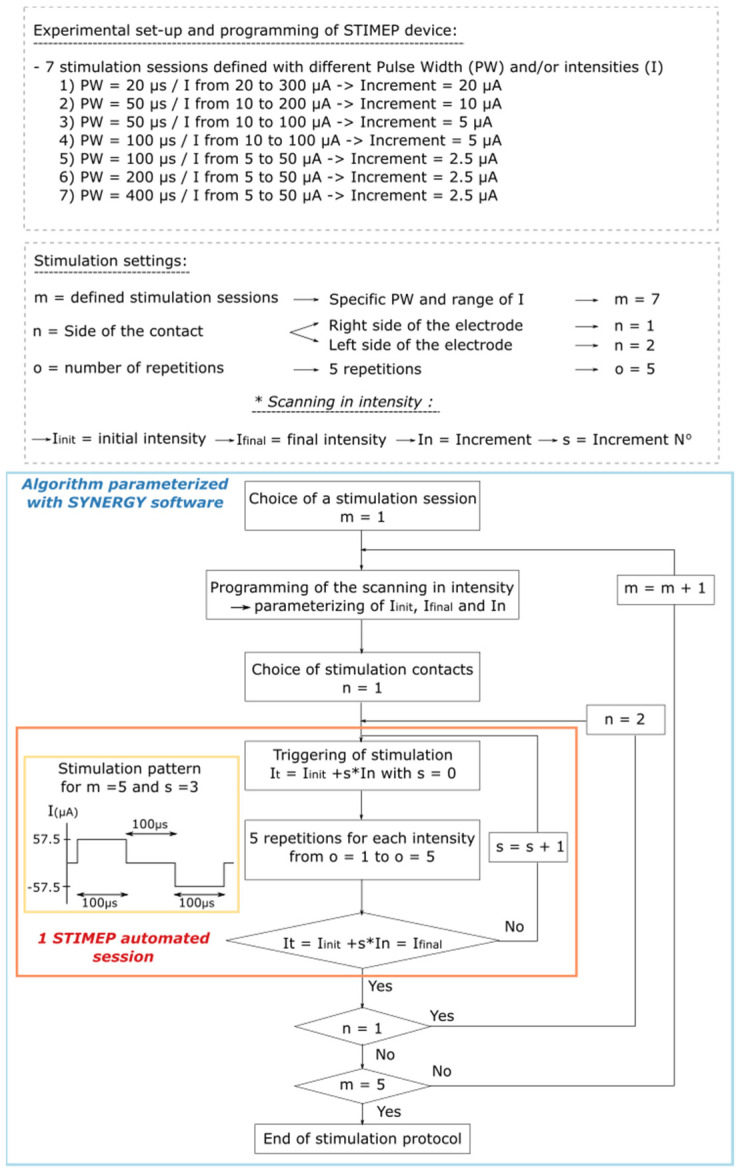
Algorithm illustrating parameterization of SYNERGY software to implement Protocol 1. Protocol 1 was designed to perform scanning in intensities with different pulse widths using a TIME implanted in a single fascicle (TIME#1). This experiment was implemented to ensure STIMEP ability to automatically deliver finely tuned stimulations but also to assess the relative impact of both intensity and pulse width in intrafascicular stimulation selectivity.

In the first rat, a single TIME was implanted in the sciatic nerve (TIME#1). The impact of the stimulation parameters on stimulation selectivity in one fascicle, i.e., the tibial fascicle, was evaluated by testing several scans of intensity which ranged from 5 to 300 µA in full scale and the pulse widths, which were 20, 50, 100, 200 and 400 µs. The scanning that was conducted using decreasing current increments—from 20 down to 2.5 µA—were parameterized to ensure STIMEP accuracy and also to identify the best stimulation parameters for both stimulation selectivity and power consumption (Figure 3).

- Protocol 2: Stimulation using two DSU simultaneously to drive two TIME implants

Two TIMEs were implanted in the sciatic nerve of the second rat. One implant (TIME#2) was connected to STIMEP′s first Distributed Stimulation Unit (DSU #1) and distally inserted while the second one (TIME#3) was implanted more proximally and was connected to the fourth DSU (DSU #4—Figure 2b). Two contacts per electrode (one on the right and one on the left side) were placed in the nerve at the same time for a total of four implanted contacts.

Both the stimulator ability to drive multi TIME implants and the relative impact of multi-TIME stimulation approaches on selectivity were investigated by using appropriate parameters for the stimulation frequencies of the two DSU. An objective comparison of muscle activities when stimulating with a single or both TIMEs was made viable by doubling the frequency of one DSU relative to the other. This resulted in synchronized pulse delivery and alternations of stimulation pulses for either one or two of the TIME implants.

Stimulations frequencies of 8 and 4 Hz were retained for multi-TIME DSU parameterizing while scanning was performed with various intensities, from 60 to 300 µA (by steps of 60 µA) with 8 and 4 repetitions respectively, symmetrical biphasic pulses—to generate recruitment curves. Two sessions were performed successively to compare the results obtained with single a TIME stimulation—with TIME#2 or TIME#3 alternatively—to those achieved when using both the DSUs. Tests were first performed with the two contacts on the right side before switching to those on the left side (Supplementary Figure S1).

- Protocol 3: Implementation of two stimulation waveforms

The versatility of STIMEP was further investigated by assessing the impact of the stimulation waveform on selectivity. A new stimulation profile was programed using the SYNERGY platform and experiments performed in protocol 2 were reproduced using an inverted stimulation profile. In other words, the original symmetrical biphasic pattern was inverted to precede the second depolarizing phase by an initial hyperpolarizing phase (Supplementary Figure S2).

#### 2.2.4. Signal Processing

EMG signals were amplified (×1000 for PL, ×100 for GM and ×200 for TA—P511AC Amplifiers, Grass Instrument Co, West Warwick, RI, USA) and band-pass filtered (5 Hz to 20 kHz for high-pass and low-pass filtering respectively) before sampling—20 ksamples/s—and their acquisition using a PowerLab system (ADInstrument, Sydney, Australia). EMG signals were first synchronized to the STIMEP trigger output before the calculation of the Root Mean Square (RMS) for a 10 ms time limit beginning 1 ms after the stimulation using Labchart software (ADInstruments, Sydney, Australia). The RMS was defined for each acquisition channel—i.e., PL, GM and TA—and each stimulation pulse before averaging for each configuration using custom software written in Matlab (Mathworks, Natick, MA, USA).

## 3. Results

### 3.1. STIMEP Successfully Delivered Finely Tuned Stimulations That Potentiate Intrafascicular Stimulation Selectivity

**Figure 4:**
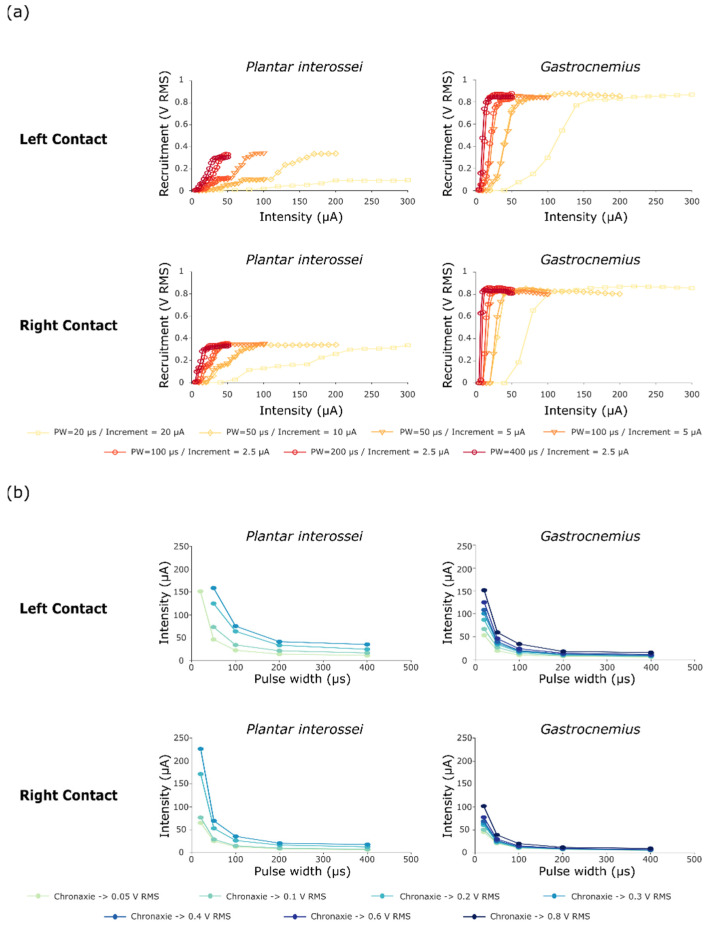
Recruitment and chronaxie curves obtained after performing protocol 1. (**a**) Recruitment curves of Plantar interossei and Gastrocnemius muscles after stimulating with either a contact on the left or on the right side of the TIME#1 implant. Muscle activity is displayed as the RMS of the compound muscle action potential. (**b**) Chronaxie-rheobase-like curves plotted as a result of these stimulations, presenting the relative recruitment of both Plantar Interossei and Gastrocnemius muscles.

The first stimulation protocol (Figure 3) aimed both to ensure the ability of STIMEP to comply with a precise microstimulation and also to investigate the impact of both the stimulation intensity and the pulse width on selectivity when implanting one TIME in a single fascicle. Data obtained after scanning in intensities were processed to obtain the RMS values. These values were then combined to plot recruitment curves (Figure 4a) and chronaxie-rheobase-like curves (Figure 4b). We focused our analysis on intrafascicular selectivity and compared the recruitment profile of two muscles innervated by the tibial fascicle—i.e., the plantar interossei and the gastrocnemius muscles [24].

Shorter pulse widths of primarily 20 µs, but also of 50 and 100 µs, increased the range between the activation threshold and the maximal recruitment for both muscles, generating a larger range whereby the level of recruitment slowly increases as a function of stimulation intensity. The recruitment curves suggested a two stage recruitment profile for the plantar interossei muscle—especially for the left stimulation contacts and the short pulse widths (Figure 4a—PW = 20 and 50 µs)—as a plateau phase was apparent at the middle of the curve at 0.10 V RMS for a full scale activation of 0.34 V RMS. After comparing plantar interossei and gastrocnemius muscle recruitment plots for a 20 µs pulse width (Figure 4a—Left contact), an increase in the selectivity range was observed as the two-stage activation of the plantar interossei muscle potentiated the recruitment gap between both muscles (Plantar interossei recruitment blocked at the plateau stage for intensities up to 300 µA—0.094 V RMS—while Gastrocnemius saturation was reached below 200 µA—0.87 V RMS). This was further confirmed by chronaxie curves presenting a very low activation of Plantar interossei (0.05 V RMS) and maximum recruitment of gastrocnemius muscle (0.8 V RMS) for medium intensities around 150 µA (Figure 4b). On the contrary, even small increments in intensity—down to 2.5 µA between two successive intensities—did not prove to lengthen the selectivity window between activation threshold and maximum recruitment when stimulating with larger pulse widths. This was highlighted by the relative superimposition of recruitment curves for several intensity steps when stimulating with 50 and 100 µs (Figure 4a).

### 3.2. STIMEP Successfully Drove Two TIME Implants Simultaneously and Increased Spatial Selectivity

**Figure 5:**
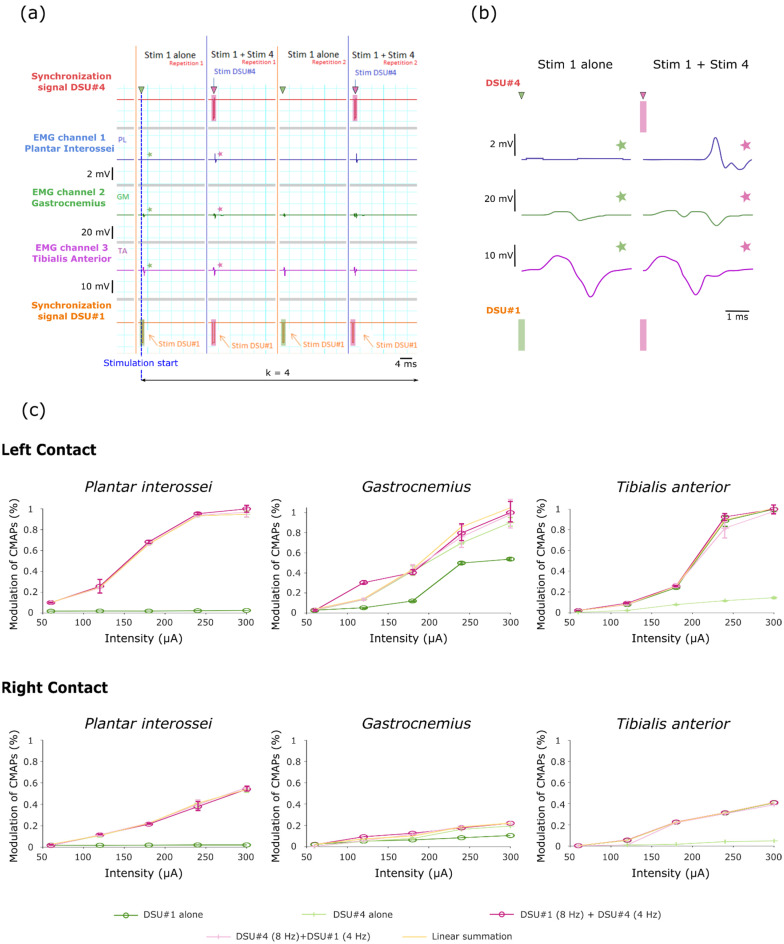
Raw signals recorded during implementation of the second stimulation protocol and corresponding recruitment curves. (**a**) Example of raw data obtained when stimulating with left contacts for a 300 µA intensity (i.e., DSU #1 = 4 Hz, DSU #4 = 8 Hz, I = 1, j = 2 and k = 4—configuration based on Supplementary Figure S1). (**b**) Close-up on the three muscles CMAPs obtained after stimulating with either a single (DSU #1) or two DSUs (DSU #1 and DSU #4)—These signals correspond to those marked with a star in (**a**). (**c**) Recruitment curves obtained for each muscle following the implementation of the stimulation protocol. Note that the “cumulated RMS T#2T#3” curve corresponds to the calculation of the RMS theoretical value obtained by linear summation of the individual responses. Similarly, the presence of two curves corresponding to the stimulation with two DSU is linked to the protocol repetition after the reversal of the stimulation frequencies on both DSUs.

Stimulations tested after the implantation of two TIMEs displayed consistently different responses, discarded the stimulation crosstalk between DSU and highlighted the reliable functionality of the STIMEP device (Figure 5a,b).

Relative positioning of the stimulation contact within the TIME implant proved crucial as the amplitude of the evoked CMAPs varied when stimulating with contacts located on the left or on the right side of the TIME implants—maximum responses over all muscles were consistently observed for the stimulation of the left contacts (Figure 5c). The raw EMG signals, evoked after the synchronous stimulation with two DSUs, revealed the relative cumulative effects as compared with the EMGs produced when stimulating with a single DSU (Figure 5a,b). The RMS values of the evoked EMGs were treated separately depending on their origin, i.e., a stimulation with one or two TIMEs and whether the stimulation was conducted using either the left or the right side contacts. For each muscle, these data were normalized by the maximum RMS value across all stimulation modalities. Recruitment curves were then plotted to characterize the effect of these stimulation modalities on muscle recruitment (Figure 5c). Using the RMS responses to single DSU stimulation (from both TIME#2 and TIME#3), a theoretical ceiling RMS value was calculated assuming the complete independence of the responses obtained following stimulation with these two TIME implants. This value was obtained by a linear summation of both TIME individual responses, by assuming the independence of the unitary responses (orange curve in Figure 5c, the Hypothesis of independence between responses evoked by stimulating with different single DSU).

The use of two contacts from different TIME implants activated all the monitored muscles while only two muscles were activated by each implant independently—TIME#2 (DSU #1) recruited the Gatrocnemius and Tibialis anterior muscle while TIME#3 activated the Gastrocnemius and the Plantar interossei. This suggests the activation of different fiber populations and an increase in the options potential movements. The recruitment profiles obtained when stimulating with both TIMEs synchronously confirm the visual impressions from the raw EMGs by presenting the cumulative effects of the individual responses (Figure 5c). Indeed, two tailed paired *t*-tests across all investigated intensities failed to display any significant differences between ceiling RMS values calculated after the linear summation of individual responses and the actual data for all the muscles and every investigated contact (t_4_ = −1.88, P = 0.13; t_4_ = 0.69, P = 0.53; t_4_ = 1.22, P = 0.29 when stimulating with left contact and t_4_ = 0.68, P = 0.53; t_4_ = 0.16, P = 0.88; t_4_ = 1.99, P = 0.11 when stimulating with right contact for Plantar interossei, Gastrocnemius and Tibialis anterior muscles respectively).

### 3.3. STIMEP Generated Complex Waveforms and Underlines the Relative Impact of Polarity on Stimulation Selectivity Using TIME Implants

**Figure 6:**
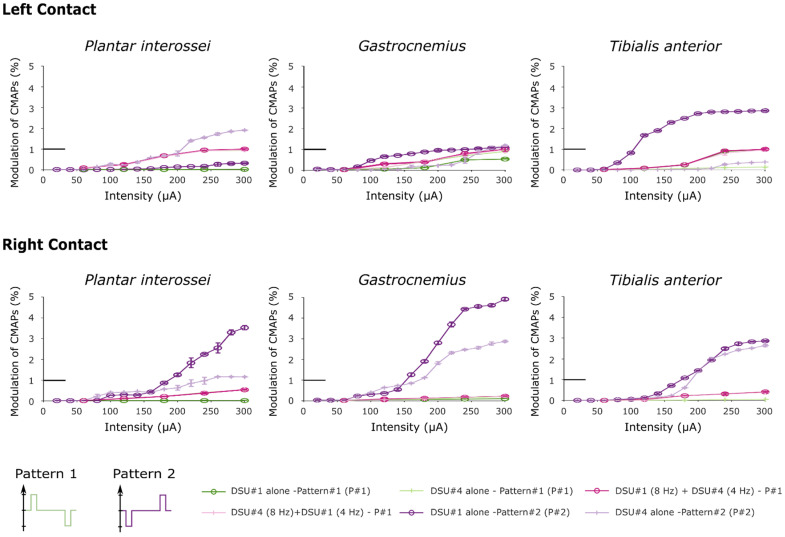
Recruitment curves obtained after stimulating with two distinct stimulation patterns. In this figure, both “Pattern 2” curves were obtained after performing the third protocol. Note that the little horizontal bold line indicates the maximum RMS value used to normalize the data (corresponds to y = 1).

The versatility of the STIMEP device was further investigated by implementing another complex stimulation waveform. This inverted biphasic profile—i.e., depolarizing pulse preceded by a hyperpolarizing phase (protocol 3—inverted polarity relative to the original biphasic stimulation profile) was also used to investigate the relative influence of the stimulation pattern on the recruitment of the monitored muscles. None of the remaining parameters (frequency, intensity and pulse width) were altered from those of the previous protocol (protocol 2). RMS values were normalized using the same maximum RMS value predefined in subsection b., and, for each muscle, recruitment curves comparing results of both protocols are compiled in Figure 6.

Recruitment curves showed a saturation for the higher RMS values when stimulating with a hyperpolarizing pre-pulse preceding the depolarizing pulse. The increase is due to inverted polarity when stimulating with TIME#2 (DSU #1) implant, and proved to be of an even higher magnitude than when stimulating with two TIMEs simultaneously with the original pattern for the Gastrocnemius and Tibialis anterior muscles (one tailed paired *t*-test across all intensities, t_4_ = 2.74, P = 0.03 and t_4_ = 3.92, P = 0.01 respectively for left contact and t_4_ = 2.26, P = 0.04; t_4_ = 2.175, P = 0.049 for right contact). In the same way, the recruitments of the Plantar interossei and the Gastrocnemius proved significantly higher when stimulating with DSU #4 using the right contact of TIME#3 (paired *t*-test across all intensities, t_4_ = 3.34, P = 0.014 and t_4_ = 2.6, P = 0.03 respectively) although the paired *t*-test failed to reveal any statistical potentiation when stimulating with the left contact (t_4_ = 1.45, P = 0.11 and t_4_ = −1.15, P = 0.84 when comparing with stimulation with two TIMEs and t_4_ = 1.47, P = 0.11 and t_4_ = −0.55, P = 0.7 respectively after comparison with the original pattern delivered by DSU #4 alone). This further highlighted more pronounced improvements after stimulating with contacts located on the right side of the electrode.

## 4. Discussion

The implemented protocols included the fine modulation of pulse width and intensity starting at 20 µs or 5 µA which gradual decreased to 5 µs and 2.5 µA respectively, the synchronized steering of two TIMEs implants and the inversion of biphasic pulses polarity revealing a fine gradual modulation of both the selectivity and the muscle recruitment with intrafascicular TIME implants.

The impact of the stimulation parameters was evaluated by performing multiple intensity series for several pulse widths in a single fascicle. Interestingly, the factor that mostly impacted selectivity was the reduction of the pulse length. For a pulse of 20 µs, a downturn of the recruitment curve was observed, enabling the finer modulation of muscular activation. In our experiments, the use of a short pulse widths efficiently widens the activation gap between plantar interossei and gastrocnemius muscles by slowing down the recruitment of the former. Although the stimulation intensity did not seem to significantly influence stimulation selectivity alone, the combination of short pulses with a low increment seems the best approach to optimize selectivity.

The implantation of two TIME devices enabled the individualization of two distinct patterns of response with one mobilizing both the plantar interossei and gastrocnemius muscles while the other recruited the tibialis anterior and the gastrocnemius. A further comparison of experimental data derived from the synchronized use of the two TIME implants with the linear summation of their unitary activities revealed no marked differences, which led to the omittance of the occurrence of nonlinear phenomenon. We thus produced an increase in the stimulation selectivity by successfully activating several independent pools of nerve fibers located in different areas of the sciatic nerve. These results proved reliable after stimulating with both the left and the right side of the TIMEs, although a clear reduction in amplitude was observed when stimulating with the right contacts. To summarise, the implantation of several TIMEs allowed for an increase in the spatial selectivity of motor fibers recruitment.

Lastly, the use of inverted polarity, including a hyperpolarizing phase before the depolarizing pulse, resulted in a potentiation of both plantar interossei and tibialis anterior recruitment without a modification of the selectivity profile—which was evident for the case the left side contacts with an activation of Pl and GM muscles for TIME#2 and of the TA and the GM for TIME#3. To provide an overview, both the added hyperpolarizing pre-pulse and the use of small pulse widths enhanced the selectivity of nerve fibers recruitment when stimulating with the TIME implants.

As previously mentioned, these results further highlight the necessity for powerful stimulation tools that allow for the implementation of complex stimulation patterns as well as an appropriate adjustment of the stimulation parameters on multiple channels/electrodes. The STIMEP platform presented in this paper meets these requirements and should ultimately allow for further refined parameters for selective stimulation.

In many ways, the results presented in this paper are consistent with previous studies modeling the peripheral nerve responses to stimulation. The effects of an initial hyperpolarizing phase and of small pulse widths had already been assessed using extraneural cuffs [14,15] and longitudinal intraneural electrodes (only two pulse widths were investigated—i.e., 20 and 50 µs [19]) but without the inclusion of transfascicular implants. Extrapolating from our results, we suggest that similar stimulation properties should be used for both intraneural (longitudinal and transversal) and extraneural electrodes. In the context of selective peripheral nerve stimulations, the results suggest the use of biphasic pulses— first charge balanced with a hyperpolarizing phase—with small pulse widths (about 20 µs) and an interphase delay of 100 µs between the two active phases [49].

Our selectivity study constitutes only a fragmented approach that is insufficient to investigate the peak potential of TIME electrodes for the selective stimulation of the peripheral nerve—approaches such as “depolarizing prepulses” [32] and “rising ramps” [33] have yet to be tested. Further, the STIMEP limitation in frequency prevents the implementation of “high frequency block” protocols [34] (Table 1) while an “anodal block” procedure is not appropriate with TIME implants as both anodes and cathodes are required to be located along the nerve fibers [31].

The implementation of symmetrical biphasic stimulation patterns may have limited data interpretation as either phase could have been responsible for muscle recruitment. The use of a small interpulse delay—100 µs as advised by Mortimer 2000 [48] and further recommended by Maciejasz et al. [49]—combined with a 20 kHz sampling frequency further precluded the analyses of the latency that could have assisted in identifying the role of hyperpolarizing prepulses as implemented in pattern#2. However, the use of symmetric charge-balanced pulses was considered to be reasonable so as to avoid nerve damage during peripheral nerve stimulation and to also maximize the range of frequency that could be delivered using STIMEP.

Only two contacts for each TIME were implanted within the rat sciatic nerve at the same time, underlining the need for further experiments in a larger animal model or for the resizing of the implant. Indeed, an increase in the number of contacts implanted transversely within the nerve favor an increase in selectivity, as previously shown in Badia et al. [24]. Furthermore, the lack of a histological study prevents the bridging of electrophysiology and anatomy.

**Figure 7:**
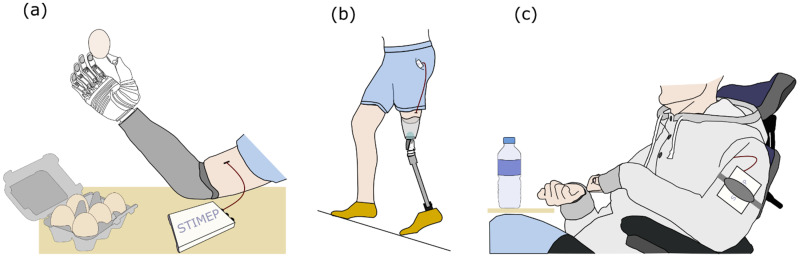
Clinical trials performed using the STIMEP Neurostimulator. (**a**) Implantation of TIME electrodes transversally in upper limb nerves and stimulation with STIMEP device to restore rich sensations in amputees [50,51,52,53]. (**b**) Combined use of TIME implants and STIMEP to optimize walking performances in lower-limb amputees [54,55]. (**c**) Restoration of upper-limb movements in individuals with spinal cord injury using multicontact cuff electrodes and STIMEP neurostimulator (ongoing clinical trial—manuscript in preparation).

Nevertheless, the rat model proved adequate to obtain a proof of concept and safety assessment but also to ensure the fulfillment of the specifications that supported the validity of the hypothesis that was tested in the aforementioned protocols; especially since the use of the final design of both the STIMEP and TIME implants allowed for the validation of the stimulation set-up prior to the implementation of a clinical trial in amputees [50,51,52,53,54,55]. During this trial, stimulating with TIME electrodes improved the richness and accuracy of the evoked sensations compared to those elicited via multicontact cuff electrodes [14,15] in upper limb amputees. Patients were thus able to “feel” the object and greatly enhance their manipulation abilities (Figure 7a) [50,51,52,53]. In the same way, the overall system—STIMEP and TIME implants—was successfully used in real-time to modulate neural stimulation in lower limb amputees when walking. In practice, these patients were able to “feel” both the ground and the knee angle of their prosthetic device which greatly improved their walking performances (Figure 7b) [54,55]. Moreover, in both cases, phantom pain significantly decreased throughout the trial. Nevertheless, these studies failed to fully investigate the potential of STIMEP for functional recovery.

The same core stimulation system was also used with complex multicontact cuff electrodes [56,57] that allow for the selective stimulation of the upper limb muscles responsible for hand and wrist movements through radial and median nerve stimulations in individuals with a spinal cord injury. STIMEP is currently being used in a second clinical trial that is ongoing (clinical trial identifier: NCT04306328—manuscript in preparation, Figure 7c), following these two preliminary experiments.

The results of this study support the versatility of this new stimulation platform. STIMEP and its associated software, SYNERGY, enabled the automated delivery of complex sequences of stimulation, the fine tuning of parameters (i.e., intensity, pulse width and frequency), the steering of up to four stimulation units independently—for a total of 56 stimulation channels—and the programming of multiple pulse waveforms. Alongside these intrinsic mechanisms, the dimensions of STIMEP make it an ideal portable stimulator for both clinical trials and ambulatory protocols.

## 5. Conclusions

In this paper, we introduce new stimulation tools and present several strategies designed to optimize the use of TIME implants in neural stimulation. By implanting several electrodes in the same nerve and tuning the stimulation parameters, we were able to validate the overall STIMEP platform and to also identify several axes of improvement and to confirm predictions from the computational studies. Among these results, the use of short pulses and the introduction of a hyperpolarizing pre-pulse seem to be of particular interest for selectivity optimization. In the same way, the implantation of several electrodes increases the size of the stimulated area and greatly enhances the overall potential of such an approach.

## Figures and Tables

**Table 1:** Technical specifications of STIMEP stimulator.

Parameter	Value
Type of stimulation	Constant current stimulation
Number of channels per stimulation unit	14 active sites (capacitively coupled) 2 references (non coupled)
Number of distributed stimulation units (DSU)	4 (56 active sites in total)
Weight Dimensions (W × L × H)	150 g 81 × 130 × 21 mm^3^
Pulse width	2–508 µs (2 µs resolution)
Intensity	10–2540 µA (10 µA resolution)
Frequency	3 ranges: low/mid/high Low range: up to 8 channels per electrode (DSU) -> 4 to 58 Hz Mid range: up to 4 channels per electrode (DSU) -> 4 to 111 Hz High range: up to 2 channels per electrode (DSU) -> 4 to 200 Hz
Passive discharge	150 µs minimum duration
Channel capacitive coupling	330 nF
Output voltage	19 V
Powering	USB or external battery
Autonomy (external battery)	8 hours

## Data Availability

Data sharing is not applicable to this article.

## Supplementary Materials

The following are available online at www.mdpi.com/article/10.3390/s21217219/s1, Figure S1: Algorithm illustrating parameterization of SYNERGY software to implement Protocol 2. Protocol 2 was designed to perform stimulation using two DSU simultaneously (DSU #1 and DSU #4) in order to drive two TIME implanted in different fascicles (TIME#2 and TIME#3). 5 stimulation intensities were tested—from 60 to 300 µA—with constant pulse width (20 µs) and multiple repetitions (either 4 or 8 repetitions for the 4 Hz or the 8 Hz stimulation). This experimental session aimed to assess STIMEP ability to deliver synchronized stimulation on different DSU but also to highlight the potential of a multi-TIME approach for rehabilitation purposes. Figure S2: Algorithm corresponding to Protocol 3. 15 stimulation intensities were tested—from 20 to 300 µA—with constant pulse width (20 µs) and frequency (5 Hz). This third protocol was designed to investigate the impact of stimulation pattern on selectivity by reversing pulses polarity—experiments were performed using the same experimental set-up as protocol 2—i.e., using two TIMEs (TIME#2 and TIME#30 connected to DSU #1 and DSU #4 respectively.
