# Supplementary material for: New Stimulation Device to Drive Multiple Transverse Intrafascicular Electrodes and Achieve Highly Selective and Rich Neural Responses
Source: Sensors (Basel). 2021 Oct 29;21(21):7219. doi: 10.3390/s21217219 (PMC8587292; doi:10.3390/s21217219)
Supplement: Supplementary file 1 [file sensors-21-07219-s001.zip › sensors-1406010-supplementary.pdf]

sensors

Article

New Stimulation Device to Drive Multiple Transverse Intrafascicular Electrodes and Achieve Highly Selective and Rich Neural Responses

Thomas Guiho [1,*], Victor Manuel López-Álvarez [2], Paul Čvančara [3], Arthur Hiairrassary [1], David Andreu [1,4], Thomas Stieglitz [3], Xavier Navarro [2] and David Guiraud [1,4]

Supplementary Figures

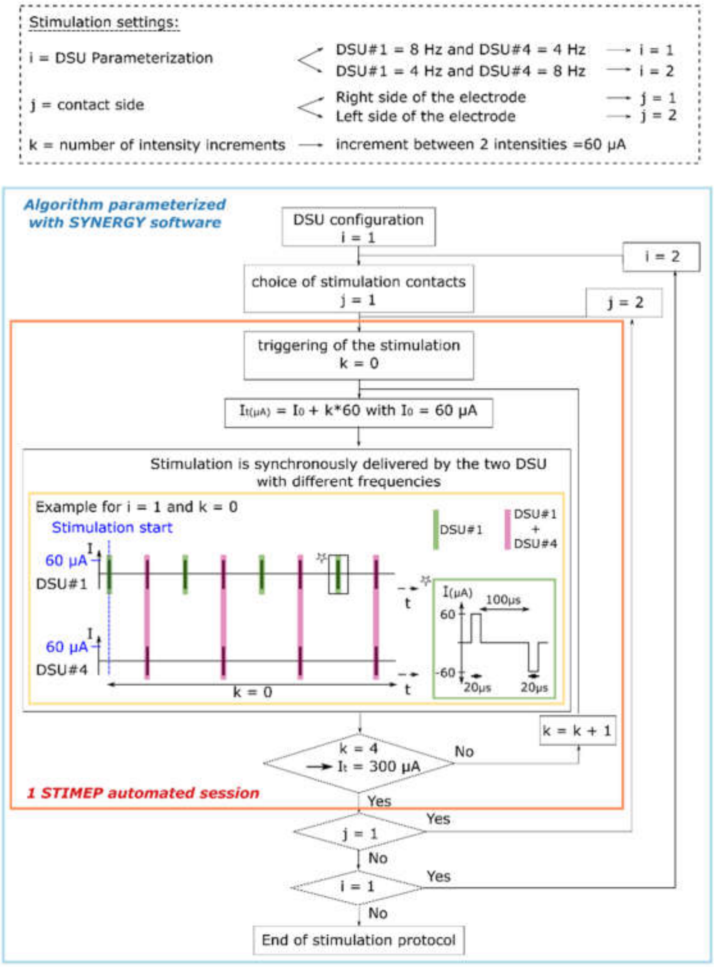

Supplementary Figure S1. Algorithm illustrating parameterization of SYNERGY software to imple-ment Protocol 2. Protocol 2 was designed to perform stimulation using two DSU simultaneously (DSU#1 and DSU#4) in order to drive two TIME implanted in different fascicles (TIME#2 and TIME#3). 5 stimulation intensities were tested from 60 to 300 μA with constant pulse width (20 μs) and multiple repetitions (either 4 or 8 repetitions for the 4 Hz or the 8 Hz stimulation). This experi-mental session aimed to assess STIMEP ability to deliver synchronized stimulation on different DSU but also to highlight the potential of a multi-TIME approach for rehabilitation purposes.

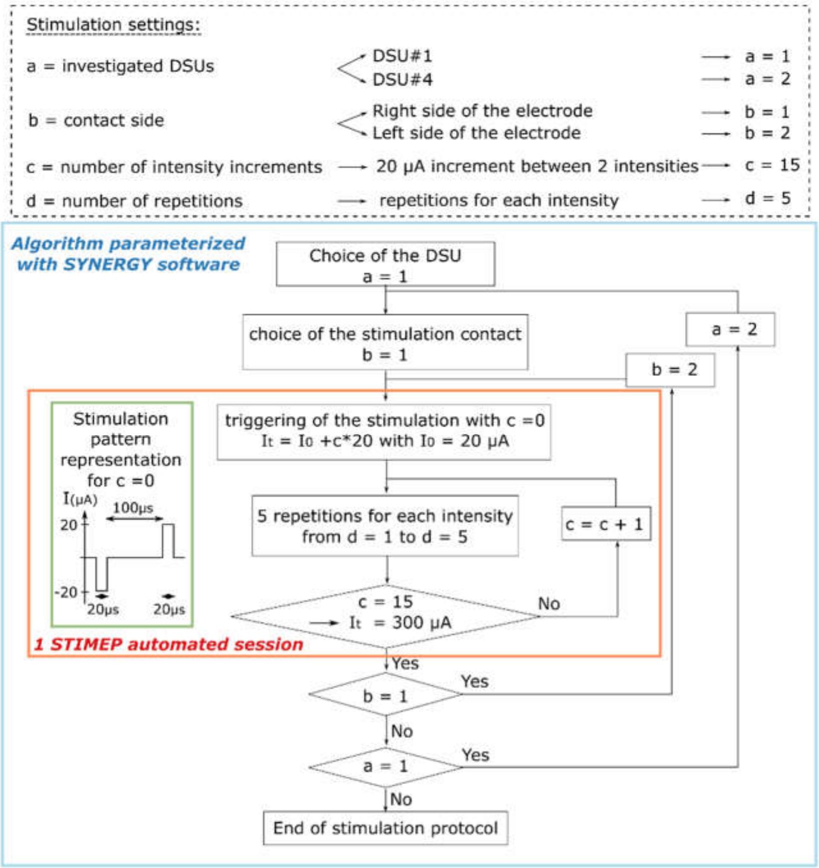

Supplementary Figure S2. Algorithm corresponding to Protocol 3. 15 stimulation intensities were tested from 20 to 300 µA with constant pulse width (20 µs) and frequency (5 Hz). This third protocol was designed to investigate the impact of stimulation pattern on selectivity by reversing pulses po-larity – experiments were performed using the same experimental set-up as protocol 2. i.e., using two TIMEs (TIME#2 and TIME#30 connected to DSU#1 and DSU#4 respectively.
